# Supplementary material for: Mapping the evolution of fertility support policies in China: A content and instrumental analysis
Source: PLoS One. 2025 Oct 9;20(10):e0332137. doi: 10.1371/journal.pone.0332137 (PMC12510515; doi:10.1371/journal.pone.0332137)
Supplement: S1 Appendix — (ZIP) [file pone.0332137.s001.zip › S1 Appendix. 226 original policy documents/184-国家发展改革委关于印发《2022年新型城镇化和城乡融合发展重点任务》的通知(FBM-CLI-4-5115091).doc]

国家发展改革委关于印发《2022年新型城镇化和城乡融合发展重点任务》的通知

**发布部门：** [国家发展和改革委员会(含原国家发展计划委员会、原国家计划委员会)](https://www.pkulaw.com/law/chl?Aggs.IssueDepartment=60301) [机构沿革](javascript:void(0))

**发文字号：**发改规划〔2022〕371号

**发布日期：**2022.03.10

**实施日期：**2022.03.10

**时效性：** [现行有效](https://www.pkulaw.com/law/chl?Aggs.TimelinessDic=01)

**效力级别：** [部门工作文件](https://www.pkulaw.com/law/chl?Aggs.EffectivenessDic=XE0303)

**法规类别：** [城市规划与开发建设](https://www.pkulaw.com/law/chl?Aggs.Category=04903)

国家发展改革委关于印发《2022年新型城镇化和城乡融合发展重点任务》的通知
（发改规划〔2022〕371号）

中央和国家机关有关部门、有关直属机构，各省、自治区、直辖市及计划单列市、新疆生产建设兵团发展改革委，全国工商联，中国国家铁路集团有限公司、国家开发银行、中国农业发展银行：
　　经城镇化工作暨城乡融合发展工作部际联席会议第四次会议审议通过，现将《2022年新型城镇化和城乡融合发展重点任务》印发给你们，请认真贯彻执行。

国家发展改革委
　　2022年3月10日

2022年新型城镇化和城乡融合发展重点任务

　　以习近平新时代中国特色社会主义思想为指导，贯彻落实中央经济工作会议精神，贯彻落实《国家新型城镇化规划（2021-2035年）》，深入推进以人为核心的新型城镇化战略，提高新型城镇化建设质量，以实际行动迎接党的二十大胜利召开。
　　一、提高农业转移人口市民化质量
　　坚持把推进农业转移人口市民化作为新型城镇化首要任务，重点针对存量未落户人口深化户籍制度改革，健全常住地提供基本公共服务制度，提高农业转移人口融入城市水平。
　　（一）持续深化户籍制度改革。各类城市要根据资源环境承载能力和经济社会发展实际需求，畅通在本地稳定就业生活的农业转移人口举家进城落户渠道。城区常住人口300万以下城市落实全面取消落户限制政策。实行积分落户政策的城市确保社保缴纳年限和居住年限分数占主要比例。鼓励人口集中流入城市区分中心城区和新区郊区等区域，制定差异化落户政策。推动具备条件的都市圈和城市群内户籍准入年限同城化累计互认。（公安部、发展改革委、各省级有关部门等负责。以下任务均需各省级有关部门落实，不再列出）

　　（二）推进城镇基本公共服务均等化。积极扩大公办学位资源，以流入地政府为主、公办学校为主，保障农民工随迁子女平等接受义务教育，落实以居住证为主要依据的随迁子女入学政策，优先将随迁子女占比较高的民办义务教育学校纳入政府购买学位范围。以新生代农民工为重点推动参保扩面，推动企业为农民工缴纳职工养老、医疗、工伤、失业、生育等社会保险费，合理引导灵活就业农民工按规定参加基本医疗保险和基本养老保险。推进异地就医跨省直接结算扩面，实现每县开通至少一家联网定点医疗机构开展门诊费用跨省直接结算。推进落实新就业形态就业人员职业伤害保障试点。有条件的地区要落实外埠老年人同等享受本地优待项目。完善基本公共服务标准体系。探索推行电子居住证。（发展改革委、教育部、财政部、人力资源社会保障部、公安部、卫生健康委、医保局等负责）

　　（三）加强农民工就业服务和技能培训。稳定和扩大农民工就业岗位，拓宽灵活就业渠道，规范平台企业用工，建设一批规范化零工市场。推进劳务品牌建设，健全劳务品牌质量体系和评价标准。清理整顿人力资源市场和劳务派遣等领域秩序，严厉打击就业歧视和非法职介等侵权行为。大力开展适合农民工就业的技能培训和新职业新业态培训，深入实施“技能中国”行动，以实用性、针对性为导向，开展农民工补贴性培训600万人次以上。（人力资源社会保障部等负责）

　　（四）健全配套政策体系。加大中央财政农业转移人口市民化奖励资金支持力度，进一步发挥中央财政市民化奖励资金激励作用。推动省级财政建立健全农业转移人口市民化奖励机制，重点支持吸纳跨市域农业转移人口落户多的城市。健全城镇建设用地增加规模与吸纳农业转移人口落户数量挂钩机制。依法保障进城落户农民的农村土地承包权、宅基地使用权、集体收益分配权，健全市场化退出机制。发布“七普”“六普”各城市城区常住人口和市域常住人口数据，做好各级行政区户籍人口城镇化率统计工作。（财政部、公安部、发展改革委、自然资源部、农业农村部、统计局等负责）
　　二、持续优化城镇化空间布局和形态
　　促进大中小城市和小城镇协调发展，推动形成疏密有致、分工协作、功能完善的城镇化空间格局。

　　（五）健全城市群一体化发展机制。积极推进京津冀协同发展，有序推进粤港澳大湾区建设，提升长三角一体化发展水平。制定出台成渝地区双城经济圈建设年度工作要点，推进一批重大项目和重大平台建设，开展经济区和行政区适度分离改革。印发实施长江中游、北部湾、关中平原城市群发展“十四五”实施方案，推动建立城市群多层次、常态化协商协调机制。有序引导其他城市群发展，深化基础设施、产业科技、生态环境、公共服务等领域合作。（发展改革委等负责）

　　（六）培育发展现代化都市圈。健全省级统筹、中心城市牵头、周边城市协同的都市圈同城化推进机制。支持有条件的都市圈科学规划多层次轨道交通，统筹利用既有线与新线发展城际铁路和市域（郊）铁路，摸排打通国家公路和省级公路“瓶颈路”，打造1小时通勤圈。支持合作共建产业园区，促进教育医疗资源共享，健全重大突发事件联防联控机制。支持有条件的都市圈探索建立税收分享和经济统计分成机制。（发展改革委、交通运输部、自然资源部、住房城乡建设部、教育部、卫生健康委、国铁集团等负责）

　　（七）促进超大特大城市优化发展。疏解非核心功能，提高健康宜居安全水平。系统运用资源环境承载能力、国土空间开发适宜性评价、城市体检结果，开展城市风险评估。中心城区人口密度高且人口持续流入的超大特大城市要有序实施功能疏解。高质量高标准推进国家级新区建设，完善郊区新城功能，推动组团式发展。（发展改革委、自然资源部、住房城乡建设部等负责）

　　（八）推进以县城为重要载体的城镇化建设。支持一批具有良好区位优势和产业基础、资源环境承载能力较强、集聚人口经济条件较好的县城发展。推进县城产业配套设施提质增效、市政公用设施提档升级、公共服务设施提标扩面、环境基础设施提级扩能，促进县乡村功能衔接互补。统筹运用中央预算内投资、地方政府专项债券、县城新型城镇化建设专项企业债券、开发性政策性及商业性金融机构信贷等资金，在不新增隐性债务前提下支持符合条件项目。加强存量低效建设用地再开发，合理安排新增建设用地计划指标。支持120个县城建设示范地区率先推动县城补短板强弱项，支持20个县城产业转型升级示范园区建设。（发展改革委、财政部、人民银行、国资委、自然资源部、住房城乡建设部、农业农村部、工业和信息化部、开发银行、农业发展银行等负责）

　　（九）完善边境地区城镇功能。推动边境城市加快发展，支持丹东、黑河、防城港等东北地区和西南地区边境地级市吸引集聚人口。开展兴边富民行动中心城镇建设试点，提高城镇综合承载能力。依托沿边公路、边民互市贸易点等，规划建设抵边新村。完善边境地区公路网络。增强边境口岸和城市的重大疫情防控救治能力，规范发热门诊建设管理。（发展改革委、交通运输部、自然资源部、住房城乡建设部、卫生健康委等负责）

　　（十）促进特色小镇规范健康发展。推动落实《[关于促进特色小镇规范健康发展意见的通知](https://www.pkulaw.com/chl/f005e57a0b8adab6bdfb.html)》及《关于印发[全国特色小镇规范健康发展导则的通知](https://www.pkulaw.com/chl/1681f59f82d15d64bdfb.html)》。健全各省份特色小镇清单管理制度，加强监测监督监管，防范处置违规行为，通报负面警示案例。（发展改革委、自然资源部、生态环境部、市场监管总局等负责）
　　三、加快推进新型城市建设
　　坚持人民城市人民建、人民城市为人民，建设宜居、韧性、创新、智慧、绿色、人文城市。

　　（十一）有序推进城市更新。加快改造城镇老旧小区，推进水电路气信等配套设施建设及小区内建筑物屋面、外墙、楼梯等公共部位维修，有条件的加装电梯，力争改善840万户居民基本居住条件。更多采用市场化方式推进大城市老旧厂区改造，培育新产业、发展新功能。因地制宜改造一批大型老旧街区和城中村。注重修缮改造既有建筑，防止大拆大建。（住房城乡建设部、发展改革委、自然资源部、商务部、农业农村部、开发银行等负责）

　　（十二）加强住房供应保障。以人口净流入的大城市为重点，扩大保障性租赁住房供给，着力解决符合条件的新市民、青年人等群体住房困难问题。保障性租赁住房主要利用农村集体经营性建设用地、企事业单位自有闲置土地、产业园区配套用地和存量闲置房屋建设，适当利用新供应国有建设用地建设。提高住宅用地中保障性租赁住房用地比例，单列租赁住房用地供应计划，主要安排在产业园区及周边、轨道交通站点附近、城市重点建设片区等区域。（住房城乡建设部、自然资源部、发展改革委等负责）

　　（十三）健全便民服务设施。探索社区综合服务设施“一点多用”，统筹设立托育服务设施、养老服务站、家政服务网点、体育健身设施、微型消防站、维修点、食堂、公共阅读空间，补齐养老服务设施短板，建设智能快件箱（信包箱）。发展社区商业，发挥个体工商户贴近社区优势，加大低成本场地安排和物流配送等配套政策帮扶力度。推动社区和易地扶贫搬迁集中安置区配套建设与人口规模相适应的幼儿园。建设以配建停车场为主、路外公共停车场为辅、路内停车为补充的城市停车系统，在人流密集的公共场所增加非机动车停放设施。加快建设充电桩，优化公共充换电设施建设布局。推动体育公园绿色空间与健身设施有机融合，打造绿色便捷的全民健身新载体。（发展改革委、住房城乡建设部、教育部、卫生健康委、民政部、商务部、自然资源部、能源局等负责）

　　（十四）开展燃气管道等老化更新改造。重点改造材质落后、使用年限较长、运行环境存在安全隐患、不符合相关标准规范规定的燃气、供水、排水、供热等老化管道及设施。开展公共供水管网漏损治理。开展电网升级改造，推动必要的路面电网及通信网架空线入地。（住房城乡建设部、发展改革委、开发银行、农业发展银行等负责）

　　（十五）健全防洪排涝设施。统筹防洪与排涝，整体提升城市洪涝灾害防御能力。推动河湖水系和生态空间治理修复，增加雨水调蓄空间。推动排水管网及泵站改造与建设，提高排水防涝能力。建设排涝通道，提高雨洪行泄能力。实施雨水源头减排工程，提高地面蓄水、渗水能力。实施防洪提升工程，降低外洪入城风险。（住房城乡建设部、自然资源部、发展改革委、水利部、应急部等负责）

　　（十六）增强抵御冲击能力。系统排查灾害风险隐患，健全灾害监测体系，提高预警预报水平，建立健全灾害预警与应急响应联动机制。完善公立医院传染病救治设施和疾控中心，推动地级及以上城市建设传染病医院或相对独立的综合性医院传染病区。采取搬迁避让和工程治理等手段，防治泥石流和滑坡等地质灾害。开展重要建筑抗震鉴定及加固改造。加强城市高层建筑、大型商业综合体等重点场所消防安全管理。开展国家城市安全风险综合监测预警工作体系建设试点。（自然资源部、应急部、住房城乡建设部、卫生健康委、发展改革委等负责）

　　（十七）提升智慧化水平。完善国土空间基础信息平台，构建全国国土空间规划“一张图”。探索建设“城市数据大脑”，加快构建城市级大数据综合应用平台，打通城市数据感知、分析、决策、执行环节。推进市政公用设施及建筑等物联网应用、智能化改造，促进学校、医院、养老院、图书馆等资源数字化。推进政务服务智慧化，提供商事登记、办税缴费、证照证明、行政许可等线上办事便利。实施中西部地区中小城市基础网络完善工程。（发展改革委、中央网信办、工业和信息化部、住房城乡建设部、自然资源部等负责）

　　（十八）推进绿色低碳发展。健全危险废弃物和医疗废弃物集中处理设施、大宗固体废弃物综合利用体系，积极稳妥推进生活垃圾分类工作。加快补齐城镇污水处理能力缺口，开展老旧破损和易造成积水内涝问题的污水管网、雨污合流制管网诊断修复更新。开展绿色生活创建行动，倡导绿色出行和绿色家庭、绿色社区建设。（住房城乡建设部、生态环境部、交通运输部、发展改革委、卫生健康委等负责）

　　（十九）加强历史文化保护传承。保护历史文化名城名镇和历史文化街区、历史建筑、历史地段，保留历史肌理、空间尺度、景观环境。加强革命文物、红色遗址、文化遗产保护，推进大遗址、石窟寺、重点文物古迹等保护利用，统筹做好整体保护、研究传承、展示利用。推动非物质文化遗产融入城市建设，鼓励建筑设计传承创新。禁止拆真建假、以假乱真，严禁随意拆除老建筑、大规模迁移砍伐老树，严禁侵占风景名胜区内土地。（住房城乡建设部、文化和旅游部、自然资源部、文物局、林草局等负责）
　　四、提升城市治理水平
　　树立全周期管理理念，提高城市治理科学化精细化智能化水平，推进城市治理体系和治理能力现代化。

　　（二十）强化空间治理。稳步推进市县国土空间规划编制工作，按照耕地和永久基本农田、生态保护红线、城镇开发边界的顺序，统筹划定落实三条控制线。制定报国务院审批城市国土空间总体规划审查工作规则，明确城市规模、开发强度、“三区三线”划定等要求。合理控制老城区开发强度，增加口袋公园、街心绿地、慢行系统等公共空间。严格限制新建建筑高度，治理“贪大、媚洋、求怪”等建筑乱象。（自然资源部、住房城乡建设部、统计局等负责）

　　（二十一）加强基层社会治理。坚持党对基层治理的全面领导，强化和巩固党建引领基层治理作用。推动社区居民委员会设立环境和物业管理委员会、公共卫生委员会，促进提高物业管理覆盖率和群众满意度。健全社区工作者职业体系，实施大学生社工计划，引导高校毕业生到社区就业创业。创新社区与社会组织、社会工作者、社区志愿者、社会慈善资源的联动机制。开展综合网络和全科网格建设，健全“社区发令、部门执行”等机制。建设一站式矛盾纠纷调处平台。（中央政法委、民政部、住房城乡建设部、教育部、卫生健康委、人力资源社会保障部等负责）

　　（二十二）优化行政区划设置。慎重从严把握撤县（市）改区，严控省会城市规模扩张，确需调整的要严格程序、充分论证。稳慎优化城市市辖区规模结构。（民政部、发展改革委等负责）
　　五、促进城乡融合发展
　　以县域为基本单元推动城乡融合发展，推进城镇基础设施向乡村延伸、公共服务和社会事业向乡村覆盖。

　　（二十三）推进城镇公共服务向乡村覆盖。积极建设城乡学校共同体，深化“县管校聘”改革，推进县域内义务教育优质均衡发展。发展普惠托育服务，构建多元化、多样化、覆盖城乡的婴幼儿照护服务体系，办好乡镇公办幼儿园。建设紧密型县域医共体，实行医保基金总额付费、结余留用，建立柔性人员上下流动机制，推动“县聘乡用、乡聘村用”。增强县级医院综合能力，通过对口帮扶、远程医疗、专科联盟等方式，推动城市优质医疗资源向县域下沉。健全县乡村衔接的养老服务网络，发展乡村互助式养老服务。（发展改革委、教育部、卫生健康委、民政部等负责）

　　（二十四）推进城镇基础设施向乡村延伸。因地制宜推动供水供气供热管网向城郊乡村和规模较大中心镇延伸，农村自来水普及率达到85%。推动县乡村（户）道路联通，促进城乡道路客运一体化。建设联结城乡的冷链物流、电商平台、农贸市场网络，建设重要农产品仓储设施和城乡冷链物流设施。推动城乡基础设施管护一体化。（住房城乡建设部、水利部、交通运输部、农业农村部、乡村振兴局、发展改革委、商务部等负责）

　　（二十五）稳步推进改革试验。稳妥扩大第二轮土地承包到期后再延长30年试点范围。积极探索实施农村集体经营性建设用地入市制度。深化农村宅基地制度改革试点，研究制定农村宅基地管理暂行办法。鼓励建立农村产权流转交易市场，推动全国联网、上下贯通。支持11个国家城乡融合发展试验区、农村改革试验区加快改革探索，总结推广典型经验。（农业农村部、自然资源部、发展改革委等负责）

　　（二十六）推进巩固拓展脱贫攻坚成果同乡村振兴有效衔接。推动乡村振兴重点帮扶县增强巩固脱贫成果及内生发展能力，逐步实现由集中资源支持脱贫攻坚向全面推进乡村振兴平稳过渡。推进易地扶贫搬迁大中型集中安置区新型城镇化建设。（乡村振兴局、发展改革委、农业农村部等负责）
　　六、组织实施

　　（二十七）强化部际协同。依托城镇化工作暨城乡融合发展工作部际联席会议制度，国家发展改革委会同有关部门加强统筹协调和指导督促。有关部门要细化制定政策措施，组织调动本系统力量扎实推进。

　　（二十八）压实地方责任。各省级发展改革委要牵头会同省级有关部门，做好组织调度和任务分解，加大资金和用地等要素保障力度。市县级政府要切实推动任务落实、政策落地、项目见效，加快取得实质性进展。

© 北大法宝：（[www.pkulaw.com](https://www.pkulaw.com/)) 专业提供法律信息、法学知识和法律软件领域各类解决方案。北大法宝为您提供丰富的参考资料，正式引用法规条文时请与标准文本核对。
欢迎查看所有[产品和服务](https://www.pkulaw.com/helps/2.html)。法宝快讯：[如何快速找到您需要的检索结果？](https://www.pkulaw.com/helps/70.html)[法宝](https://www.pkulaw.com/helps/69.html)V6有何新特色？

扫描二维码阅读原文

**原文链接：**<https://www.pkulaw.com/chl/1b71014ab0dc5787bdfb.html>
